# ADVANCING FOOD EQUITY AMONG OLDER ADULTS AND ADULTS WITH DISABILITIES IN THE US

**DOI:** 10.1093/geroni/igad104.0349

**Published:** 2023-12-21

**Authors:** Laura Samuel, Boeun Kim

## Abstract

Older adults and adults with disabilities face challenges related to food insecurity, defined as limited or uncertain access to adequate food due to economic and social conditions. Adults with disabilities have triple the risk of food insecurity than their peers and rates of food insecurity have been rising over the past two decades among older adults and food insecurity is known to be a risk factor for numerous adverse health outcomes. Despite knowledge of disparities, research is needed to identify and test strategies for addressing food insecurity disparities. This symposium will examine mechanisms that may contribute to food insecurity, including local food environments, vision impairment and access to the Supplemental Nutrition Assistance Program, which is the largest food assistance program in the U.S.. National data is used in all three studies to allow population-based inferences to be drawn from study findings. Results have potential to inform our understanding of food equity and advance data-driven policy and programmatic decisions.

